# Elevated serum IgG4 and eosinophilia in a persistent indeterminate biliary stricture with high-risk features: a case report

**DOI:** 10.3389/fmed.2026.1863266

**Published:** 2026-07-06

**Authors:** Ruohan Yu, Jing Zhang, Sheng-Guang Li, Tong Zhang, Lina Zhang, Yadan Zou, Ting Long, Yanfeng Zhang, Ji Li

**Affiliations:** 1 Department of Rheumatology and Immunology, Peking University International Hospital, Beijing, China; 2 Department of Pathology, Peking University International Hospital, Beijing, China

**Keywords:** CA19-9, case report, cholangiocarcinoma, elevated serum IgG4, eosinophilia, eosinophilic cholangitis, IgG4-related sclerosing cholangitis, indeterminate biliary stricture

## Abstract

**Background:**

Indeterminate biliary strictures remain diagnostically challenging because inflammatory biliary disease, premalignant epithelial change, and cholangiocarcinoma may share overlapping clinical, radiologic, serologic, and histologic features. Eosinophilic cholangitis is a rare benign inflammatory entity that can mimic malignancy, whereas elevated serum IgG4 and eosinophilia may coexist with other biliary disorders.

**Case presentation:**

A 68-year-old man presented with progressive jaundice, cholestatic liver injury, marked eosinophilia, elevated CA19-9, elevated IgE, and elevated serum IgG4. Baseline MRCP, ultrasound, and contrast-enhanced CT showed an indeterminate common bile duct stricture/occupying lesion with upstream biliary dilatation. Exploratory laparotomy with bile duct exploration revealed distal common bile duct stenosis, flocculent material, and multiple intrabiliary polypoid lesions. Pathology from the polypoid biliary lesion showed chronic inflammatory change with lymphoplasmacytic infiltration and scattered eosinophils, negative IgG4 staining, Ki-67 hot spot of approximately 10%, and wild-type p53. The distal common bile duct biopsy showed moderate chronic inflammation, compressed glands, mild cytologic atypia, negative tissue IgG4, and an epithelial Ki-67 hot spot of approximately 50%, but the sampled tissue was insufficient for a diagnosis of carcinoma. During reassessment, MRI showed a hilar nodule and lymphadenopathy, and dual-phase FDG PET/CT showed hilar/biliary uptake with an early SUVmax of 3.9 and delayed SUVmax of 5.0. Methylprednisolone 40 mg/day was started on December 28, 2019. Peripheral eosinophilia, liver biochemistry, serum IgG4, and CA19-9 improved over time, but T-tube cholangiography on January 13, 2020 still showed persistent segmental common bile duct stenosis with upstream ductal dilatation. Later family follow-up indicated clinical deterioration and an outside-hospital diagnosis of advanced biliary malignancy, but outside pathology and cause-of-death documentation were unavailable.

**Conclusion:**

This case is best interpreted as a persistent indeterminate biliary stricture with high-risk features rather than as proven eosinophilic cholangitis, IgG4-related sclerosing cholangitis, or pathologically confirmed cholangiocarcinoma. Improvement in eosinophils, serum IgG4, liver tests, or CA19-9 did not establish a benign diagnosis. Persistent anatomical stenosis after corticosteroid exposure should prompt renewed hepatobiliary malignancy assessment and repeat targeted tissue acquisition.

## Introduction

Indeterminate biliary strictures remain a difficult diagnostic problem because benign inflammatory disease, biliary epithelial neoplasia, and cholangiocarcinoma can present with overlapping clinical, serologic, radiologic, and histologic findings (1–4). Eosinophilic cholangitis (EC) is a rare benign inflammatory disorder characterized by eosinophilic infiltration of the biliary tract and may appear radiologically similar to cholangiocarcinoma (5–7). IgG4-related sclerosing cholangitis (IgG4-SC), primary sclerosing cholangitis (PSC), and obstructive cholangitis may further complicate the differential diagnosis (8–10).

The diagnostic challenge increases when a patient shows a steroid-responsive inflammatory phenotype but also has persistent structural biliary disease. Elevated serum IgG4 does not establish IgG4-related disease, and serum IgG4 elevation has been described in a subset of patients with cholangiocarcinoma (8, 9, 11). CA19-9 may rise in biliary obstruction or cholangitis and therefore cannot be interpreted independently of the obstructive context (12). Proposed EC features include biliary wall thickening or stenosis, histopathological eosinophilic infiltration, and reversibility of biliary abnormalities either spontaneously or after corticosteroid treatment (6, 13, 14).

We report a patient with marked eosinophilia, elevated serum IgG4, inflammatory biliary pathology, mild epithelial atypia, and a high Ki-67 hot spot in the distal common bile duct biopsy. The case illustrates the importance of distinguishing laboratory improvement from anatomical resolution in the evaluation of an indeterminate biliary stricture.

## Case presentation

### Patient information and initial presentation

A 68-year-old man presented in October 2019 with approximately 1 month of progressive jaundice and dark urine after a preceding period of abdominal distension and anorexia. He had no known inflammatory bowel disease, asthma, vasculitis, viral hepatitis, alcohol use disorder, smoking history, relevant family history, or known drug/food allergy history.

Initial laboratory testing on October 16, 2019 showed alanine aminotransferase (ALT) 613 U/L, aspartate aminotransferase (AST) 608 U/L, alkaline phosphatase (ALP) 1164 U/L, gamma-glutamyl transferase (GGT) 563 U/L, total bilirubin 58 μmol/L, and direct bilirubin 43.9 μmol/L. On October 22, the white blood cell count was 9.53 × 10^9^/L, eosinophils were 63.7% (absolute eosinophil count 6.07 × 10^9^/L), hemoglobin was 111 g/L, platelets were 147 × 10^9^/L, albumin was 31.9 g/L, CA19-9 was 107.1 U/mL, IgG was 16.15 g/L, IgE was 370 IU/mL, and serum IgG4 was 6.41 g/L. Antinuclear antibody was positive at 1:160 with a cytoplasmic granular pattern. Antimitochondrial antibody and AMA-M2 were negative. Anticardiolipin IgA and β2-glycoprotein I IgA were positive. ANCA and anti-GBM antibodies were negative.

### Imaging and operation

**Figure 1:**
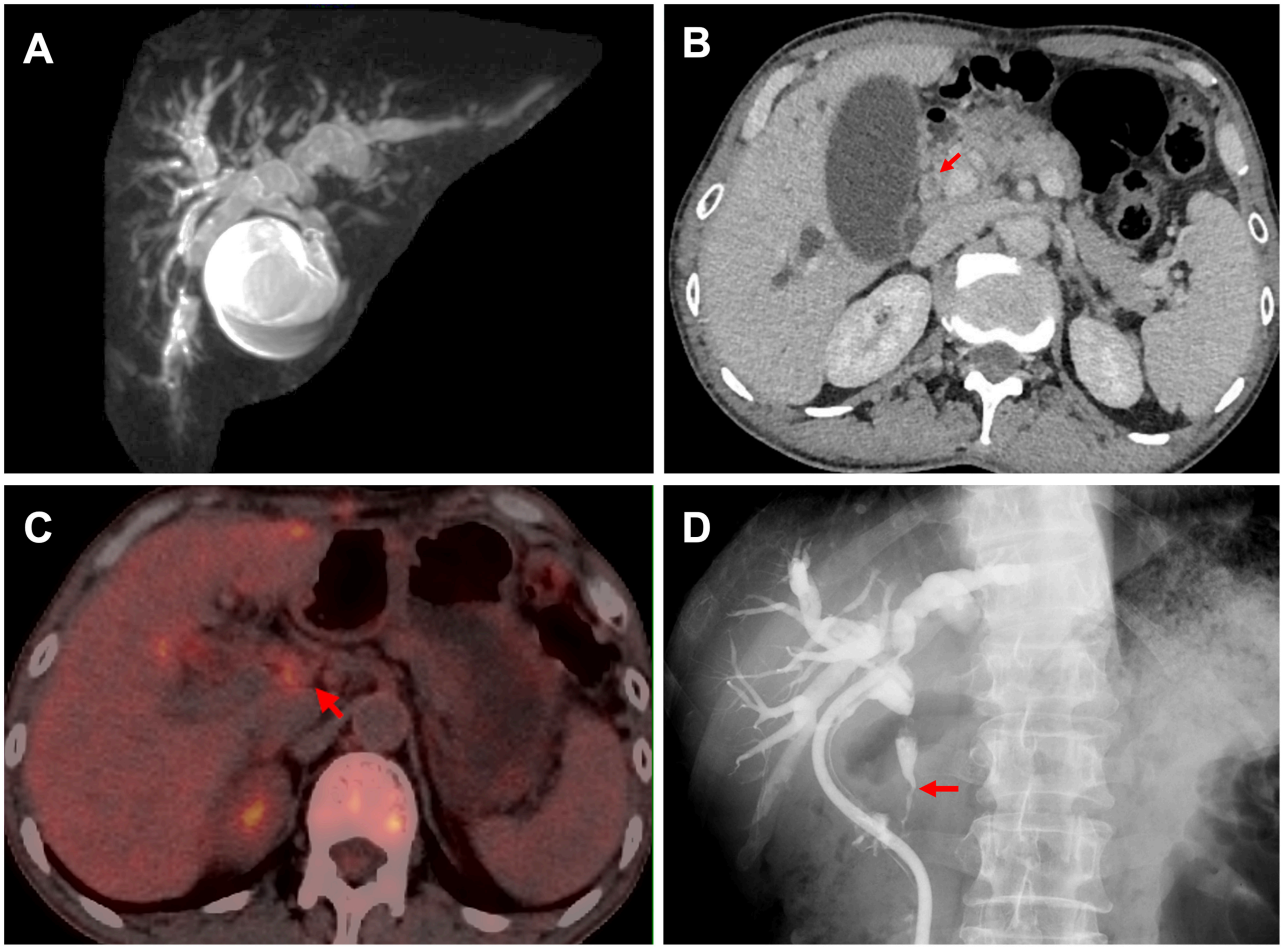
Multimodal imaging findings of the indeterminate biliary stricture. (**A**) Baseline MRCP showing marked intrahepatic bile duct dilatation; the imaging report described focal wall thickening and focal luminal narrowing in the mid common bile duct. (**B**) Contrast-enhanced abdominal CT showing a focal stenotic or mass-like lesion involving the mid-to-distal common bile duct region with upstream biliary dilatation (red arrow). (**C**) FDG PET/CT showing increased uptake in the hilar/biliary region; the early SUVmax was 3.9 and the delayed SUVmax was 5.0 (red arrow). (**D**) T-tube cholangiography after corticosteroid exposure showing persistent segmental common bile duct stenosis with marked hilar and intrahepatic bile duct dilatation (red arrow).

MRCP on October 16, 2019, showed focal wall thickening and focal luminal narrowing in the mid common bile duct, marked dilatation of the proximal common bile duct and intrahepatic ducts, and gallbladder enlargement (Figure 1A). Ultrasound described multiple bead-like soft solid nodules in the extrahepatic bile duct from the confluence of the left and right hepatic ducts to the distal common bile duct, with an involved length of approximately 4.8 cm and the largest lesion measuring approximately 1.5 × 1.2 cm. Contrast-enhanced abdominal CT showed a lower common bile duct occupying lesion with obstructive intrahepatic and extrahepatic biliary dilatation (Figure 1B).

On October 31, 2019, exploratory laparotomy, cholecystectomy, and bile duct exploration were performed. The gallbladder measured approximately 12 × 5 × 4 cm with a thickened wall. The common bile duct was enlarged, with a proximal diameter of approximately 1.1 cm. Choledochoscopy through the common bile duct incision showed distal common bile duct stenosis, flocculent material attached to the lower common bile duct mucosa, black stone residue, and multiple flocculent/polypoid lesions in the left and right hepatic ducts and branches. Biopsies were taken from the stenotic distal common bile duct and from intrabiliary polypoid tissue. Frozen sections showed inflammatory change without malignancy. Stones and flocculent material were removed, a T-tube was placed, and postoperative bile drainage was approximately 400–500 mL/day.

### Histopathological findings

**Figure 2:**
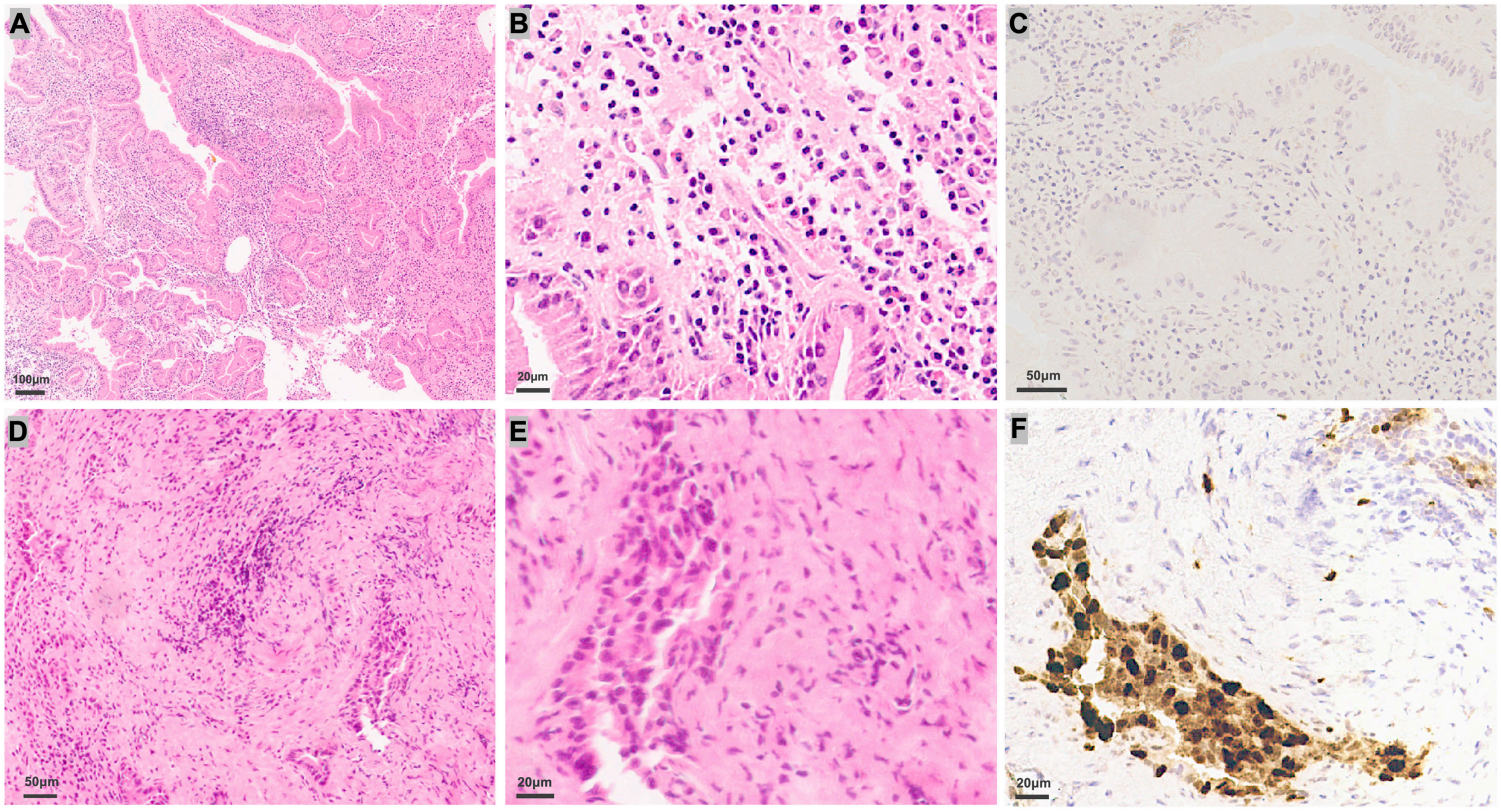
Histopathological and immunohistochemical findings. (**A**) Low-power hematoxylin and eosin staining of the polypoid biliary lesion sampled during bile duct exploration, showing papillary/polypoid inflammatory proliferation. (**B**) Higher-power view showing lymphoplasmacytic inflammation with scattered eosinophils. (**C**) IgG4 immunostaining showing negative or rare IgG4-positive plasma cells despite elevated serum IgG4. (**D**) Low-power hematoxylin and eosin staining of the distal common bile duct biopsy showing small fragments of bile-duct-wall tissue with chronic inflammation and fibrotic stroma. (**E**) Higher-power view showing compressed ductal glands with mild epithelial atypia in an inflamed fibrous background; unequivocal invasive carcinoma was not identified in the sampled tissue. (**F**) Ki-67 immunostaining of the distal common bile duct biopsy showing a high epithelial hot spot in atypical/compressed glandular structures, corresponding to the reported hot-spot index of approximately 50%. Scale bars are shown in each panel.

Permanent pathology showed chronic cholecystitis. The polypoid biliary specimen measured 2 × 2 × 0.2 cm and showed severe chronic mucosal inflammation with lymphoplasmacytic infiltration and scattered eosinophils. Immunohistochemistry showed CD138 positivity, CD38 positivity, scattered CD68-positive cells, negative IgG4 staining, partial IgG positivity, a Ki-67 hot spot of approximately 10%, and wild-type p53 expression (Figure 2A–C).

The distal common bile duct biopsy consisted of small bile-duct-wall fragments with moderate chronic inflammation, compressed relatively regular glands, and mild cytologic atypia. The morphology was regarded as insufficient for a diagnosis of carcinoma in the sampled tissue. Immunohistochemistry showed CK, CK7, and CK19 positivity, wild-type p53 expression, and an epithelial Ki-67 hot spot of approximately 50%. Repeat immunohistochemistry showed CD38 positivity, occasional CD138-positive cells, negative IgG4 staining, and weak scattered IgG staining. No definite desmoplastic stromal invasion, lymphovascular invasion, or perineural invasion was identified in the available biopsy fragments (Figure 2D–F).

### Postoperative course and subsequent evaluation

After discharge with the T-tube, clamping was followed by recurrent liver-test elevation. During reassessment, ALT and AST were reported as approximately 296 U/L and 192 U/L, respectively. On December 11–12, MRI showed a 2.6 × 2.2 cm hilar nodule, intrahepatic bile-duct dilatation, and hilar lymphadenopathy. FDG PET/CT on December 23 showed strip-like/linear increased uptake in the hilar/biliary region with an early SUVmax of 3.9 and a delayed SUVmax of 5.0 (retention index approximately 28.2%); mildly avid hilar lymph nodes were also reported (Figure 1C).

**Figure 3:**
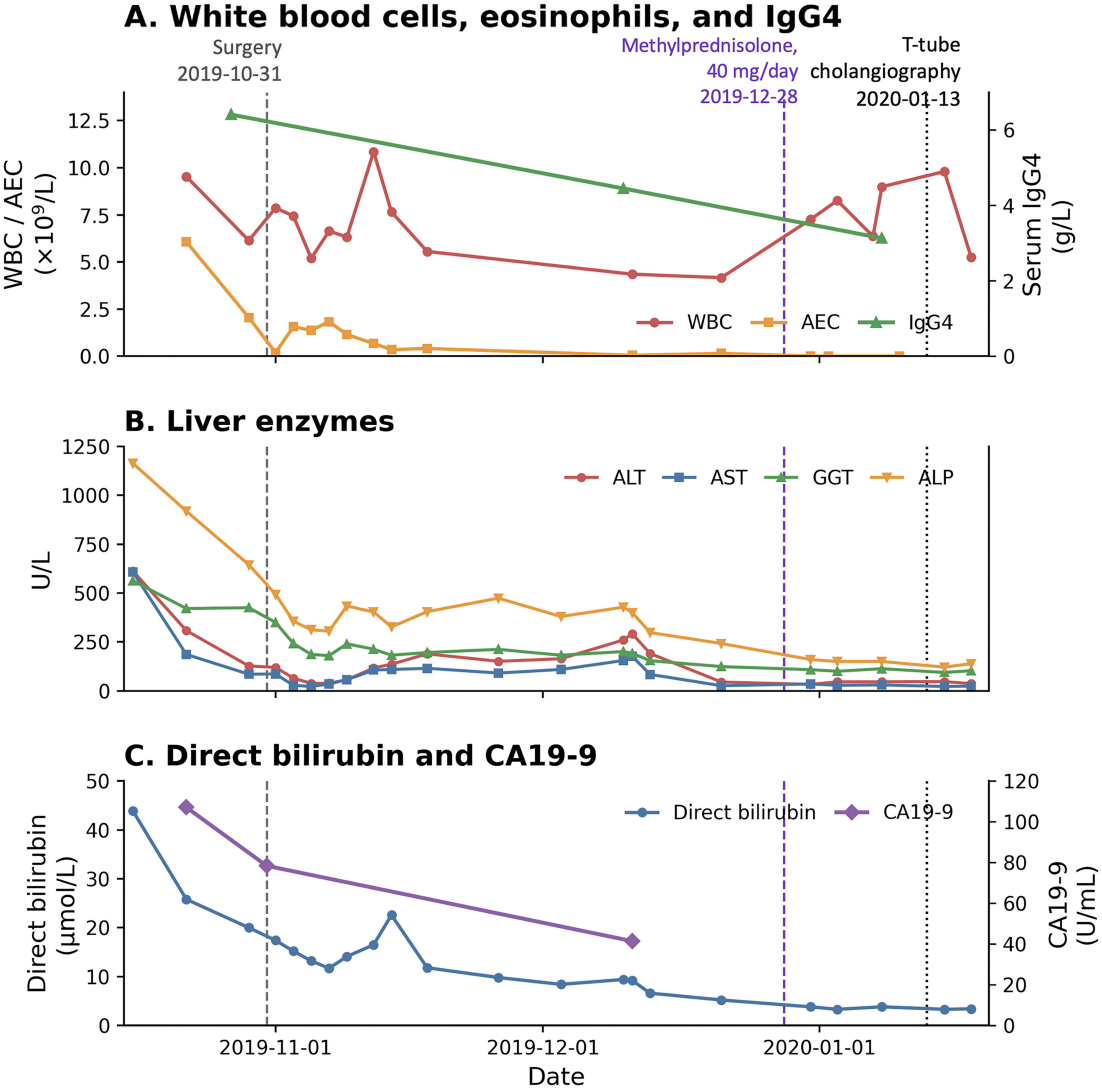
Laboratory course before and after surgery and corticosteroid treatment. (**A**) Serial white blood cell count (WBC), absolute eosinophil count (AEC), and serum IgG4 levels. (**B**) Serial liver enzyme levels, including ALT, AST, GGT, and ALP. (**C**) Serial direct bilirubin and CA19-9 levels. The vertical reference lines indicate surgery on October 31, 2019, methylprednisolone 40 mg/day started on December 28, 2019, and T-tube cholangiography on January 13, 2020. Serial serum IgG4 values were 6.41 g/L on October 27, 2019, 4.45 g/L on December 10, 2019, and 3.13 g/L on January 8, 2020. CA19-9 values were 107.1 U/mL on October 22, 2019, 78.4 U/mL on October 31, 2019, and 41.4 U/mL on December 11, 2019.

Serial laboratory trends are summarized in Figure 3. In the blood-count and immunologic panel, peripheral eosinophilia decreased over time, and serum IgG4 decreased from 6.41 g/L on October 27 to 4.45 g/L on December 10 and 3.13 g/L on January 8 (Figure 3A). In the liver-enzyme panel, ALT, AST, GGT, and ALP fluctuated after surgery and subsequently decreased during continued biliary drainage, supportive care, and corticosteroid exposure (Figure 3B). In the bilirubin and tumor-marker panel, direct bilirubin decreased over time, and CA19-9 decreased from 107.1 U/mL on October 22 to 78.4 U/mL on October 31 and 41.4 U/mL on December 11 (Figure 3C). On December 31, 2019, a case conference considered inflammatory and neoplastic causes of the biliary stricture.

### Therapeutic intervention and follow-up

Methylprednisolone 40 mg/day was started on December 28, 2019. During treatment, appetite improved and liver biochemistry decreased, although cholestatic enzymes remained abnormal. Ursodeoxycholic acid was added for persistent cholestatic enzyme elevation. By January 2, 2020, eosinophils were 0.1% and the absolute eosinophil count was 0.01 × 10^9^/L.

An ultrasound-guided liver biopsy was performed on January 7, 2020, and the specimen was sent to an outside pathology department. The complete formal pathology report and slides were unavailable for review. Despite symptomatic and laboratory improvement, T-tube cholangiography on January 13, 2020, showed persistent segmental common bile duct stenosis with hilar and intrahepatic duct dilatation (Figure 1D). He was discharged on oral prednisone 40 mg daily with advice for further hepatobiliary follow-up.

An outpatient note dated March 23, 2020 documented bile leakage around the T-tube for 4 days without fever. MRCP and chest CT were ordered, but no corresponding completed reports were available. Later family telephone follow-up indicated progressive clinical deterioration with cachexia within approximately 6 months and an outside-hospital diagnosis of advanced biliary malignancy. Outside pathology, imaging, and cause-of-death documentation were unavailable.

## Discussion

This case illustrates the diagnostic tension between an inflammatory biochemical phenotype and a structurally persistent biliary lesion. The patient had marked eosinophilia, elevated serum IgG4, elevated IgE, inflammatory biliary histology, and improvement in laboratory indices over time (Figure 3). However, he also had a dominant common bile duct stricture, a later hilar nodule, persistent anatomic obstruction on T-tube cholangiography, mild epithelial atypia in the distal common bile duct biopsy, and a markedly elevated epithelial Ki-67 hot spot (Figures 1 and 2). Taken together, the case is most appropriately interpreted as a persistent indeterminate biliary stricture with high-risk features.

The initial inflammatory differential diagnosis included EC and IgG4-SC. Published EC reports emphasize its ability to mimic cholangiocarcinoma and to respond to corticosteroids or budesonide (5–7, 13, 15, 16). Diagnostic discussions of EC have also emphasized the importance of biliary reversibility rather than biochemical improvement alone (6, 13, 14). In the present case, peripheral eosinophilia was striking and scattered eosinophils were reported in biliary tissue, but the pathology reports were qualitative rather than quantitative. More importantly, anatomic reversibility of the dominant stricture was not demonstrated. Similarly, although serum IgG4 was elevated, tissue IgG4 staining was repeatedly negative in the available biliary specimens, and there was no established multi-organ pattern to support definite IgG4-related disease (8, 9, 11). These features argue against over-classifying the case as either confirmed EC or established IgG4-SC.

The distal common bile duct biopsy is a key source of concern. Morphologically, the sampled fragments were limited and did not show definitive invasive carcinoma. Nevertheless, the presence of mild epithelial atypia together with an epithelial Ki-67 hot spot of approximately 50% is difficult to dismiss as a purely reactive change. Biliary intraepithelial neoplasia is diagnosed morphologically, and proliferation markers alone cannot establish carcinoma (17). However, the discordance between limited morphology and marked proliferative activity is clinically important: it does not prove carcinoma, but it should increase suspicion for an under-sampled high-grade intraepithelial or invasive process and should prompt further targeted tissue acquisition.

The radiologic course also remained concerning. Baseline MRCP, ultrasound, and CT all indicated a dominant obstructive lesion, and MRI later showed a 2.6 × 2.2 cm hilar nodule with hilar lymphadenopathy (Figure 1A and B). Dual-phase FDG PET/CT showed early and delayed SUVmax values of 3.9 and 5.0, corresponding to a retention index of approximately 28.2% (Figure 1C). Dual-time-point FDG PET has been used to help differentiate malignant from inflammatory processes, although overlap remains substantial and PET/CT cannot substitute for histology (18). In this case, the delayed uptake pattern should be regarded as a red flag rather than as reassuring evidence of a benign lesion.

CA19-9 also requires contextual interpretation. The decrease from 107.1 to 41.4 U/mL paralleled drainage and improvement in cholestasis (Figure 3C), consistent with evidence that CA19-9 may be elevated in both benign and malignant obstructive jaundice (12). Thus, the CA19-9 trajectory reduced the specificity of this marker as a malignancy signal but did not resolve the central problem of persistent anatomic stenosis.

The practical implication is that persistent stenosis after corticosteroid exposure should reopen the hepatobiliary malignancy pathway. Current endoscopic guidance supports active tissue acquisition for biliary strictures of undetermined etiology, including brush cytology plus fluoroscopy-guided biopsy, cholangioscopy-directed biopsy, and EUS-guided sampling when appropriate (1, 2). ERCP-based brushings and forceps biopsies are widely available but have limited sensitivity. Digital cholangioscopy can improve endoluminal visualization and enable targeted biopsies directly from suspicious mucosal areas (19). EUS-guided fine-needle aspiration or biopsy may be particularly useful when there is a mass-like lesion adjacent to the bile duct or enlarged regional lymph nodes (20). The optimal next step depends on lesion location, procedural expertise, and local availability, but the present case suggests that continued observation after laboratory improvement is insufficient when anatomic stenosis persists.

Cholangiocarcinoma often presents late and may be difficult to establish pathologically in stricturing biliary disease, particularly when sampling is limited (3, 4). PSC is also clinically relevant because dominant strictures in PSC require careful malignancy surveillance, although this patient did not have classic PSC features or inflammatory bowel disease (10). These broader hepatobiliary principles support a cautious approach to immune-mediated labels when structural obstruction and epithelial/proliferative abnormalities coexist.

This report has several limitations. Outside-hospital pathology, imaging, and cause-of-death documentation were not available, so pathologically confirmed cholangiocarcinoma cannot be claimed. Quantitative pathology data were incomplete, including eosinophil counts per high-power field, tissue IgG4-positive plasma-cell counts, and tissue IgG4/IgG ratios. Detailed archived radiologic parameters, including diffusion restriction, enhancement-phase behavior, and calibrated pre/post cholangiographic measurements, were unavailable. The complete formal pathology report and slides from the January 2020 liver biopsy were also unavailable. These limitations justify a cautious interpretation, but they do not negate the main clinical lesson: improvement in inflammatory markers did not establish benign disease in the presence of a persistent dominant biliary stricture.

## Conclusion

In patients with an indeterminate biliary stricture, marked eosinophilia and elevated serum IgG4 may coexist with high-risk epithelial and radiologic findings. Improvement in eosinophils, serum IgG4, CA19-9, and liver tests should not be equated with resolution of the lesion. Persistent anatomical stenosis after corticosteroid exposure should trigger renewed consideration of biliary neoplasia and repeat targeted tissue sampling.

## Data Availability

The original contributions presented in the study are included in the article; further inquiries can be directed to the corresponding author.
